# Enhancement of Organic Matter Removal in an Integrated Biofilm-Membrane Bioreactor Treating High-Salinity Wastewater

**DOI:** 10.1155/2018/2148286

**Published:** 2018-08-29

**Authors:** Yan Yang, Zhiyu Shao, Jun Du, Qiang He, Hongxiang Chai

**Affiliations:** ^1^Key Laboratory of the Three Gorges Reservoir Region's Eco-Environment, Ministry of Education, School of Urban Construction and Environmental Engineering, Chongqing University, Chongqing 400045, China; ^2^National Centre for International Research of Low-Carbon and Green Buildings, Chongqing University, Chongqing, China

## Abstract

High salinity can strongly inhibit microbial activity and decrease the sedimentation ability of activated sludge. The combination of biofilm and membrane bioreactor is a practical approach towards effective removal of pollutants and low fouling rate. An integrated biofilm-membrane bioreactor (BMBR) treating mustard tuber wastewater was investigated. An average COD removal efficiency of 94.81% and ammonium removal efficiency of 96.84% were achieved at an organic load of 0.5 kg COD/(m^3^·d). However, the reactor showed a relatively low efficiency in total nitrogen and soluble phosphorus removal due to the lack of anaerobic environment. The increase of influent organic load resulted in a performance degradation because a balance between the degradation ability and pollution has been reached. Images of scanning electron microscopy revealed that halophilic bacteria were the dominant microbe in the system that leads to a loose sludge structure and declined settling properties. It was found that membrane fouling was the consequence of the interaction of microbial activities and NaCl crystallization.

## 1. Introduction

There are a number of mustard tuber pickling plants in the Three Gorges reservoir watershed, which is one of the most important fresh water resources in China. These pickling plants play an important role in the local economic development. However, serious environmental pollution has emerged due to illegal discharge of the mustard tuber wastewater, which is characterized by high salinity, high nitrogen and phosphorus level, and high organic load. Direct discharge of this type of wastewater has a detrimental impact on the ecosystem, e.g., eutrophication, dehydration and death of biological cells, and changes in biodiversity [1].

Currently, treatment of high-salinity wastewater mainly includes two methods: biological treatment and physicochemical treatment. Compared with biological treatment, the physicochemical method cannot effectively remove dissolved organic matter and requires a high level of pretreatment [2]. The operational cost is so high that the wide application of the physicochemical method is prohibited. Therefore, further researches were undertaken to seek for an efficient biological process to treat high-salinity wastewater [3, 4].

It has been reported that high salinity can strongly inhibit microbial activity [5] and decrease the sedimentation ability of activated sludge [6]. Hence, it challenges the system stability and results in a low treatment efficiency. In order to overcome these difficulties, application of membrane biological reactor (MBR) to treat high-salinity wastewater has been investigated. With the advantage of membrane filtration, MBR allows more biomass to be maintained in the reactor and could achieve a complete separation of hydraulic retention time and sludge retention time [7]. Gaetano et al. [8] reported that membrane bioreactor showed high removal efficiencies under the condition of normal salinity. However, the increase of salinity significantly promoted the soluble microbial products leading to membrane fouling. Other studies showed similar results [9–11]. Membrane fouling still represents one of the major drawbacks for MBRs [12, 13]. This problem is further aggravated when they are used to treat high-salinity wastewater because microbial community characteristics play an important role in biofouling [14].

The moving bed biofilm reactor-membrane bioreactor (MBBR-MBR), proposed by Leiknes and Ødegaard [15], has been considered to be an effective biological process to mitigate the biofouling in MBR systems. Biofilm can immobilize microbes and increase the biomass concentration while the membrane separates the suspended solids and sludge. Comparative studies of the performance between MBBR-MBR and MBR have been conducted. It has been proved that the degree of membrane fouling for MBBR-MBR was far lower than that for MBR [16, 17]. Daniele et al. [18] tested the impacts of salinity on the performance of MBBR-MBR. Results indicated that the gradual salinity increase helped the acclimation of biomass, but biofilm detachments from carriers led to the irreversible cake deposition. To our knowledge, there are few studies using MBBR-MBR to treat wastewater containing high-concentration salinity, organic matter, and nutrient. The mechanism of biofouling in MBBR-MBR when treating such wastewater is still unclear.

In this context, an integrated biofilm-membrane biological reactor (BMBR) was established to treat mustard tuber wastewater in our study. The objective of the study includes (i) evaluate the performance of BMBR treating high-salinity wastewater; (ii) investigate the effect of organic load on the removal efficiency of BMBR; and (iii) explore the role of halophilic bacteria in membrane fouling.

## 2. Materials and Methods

### 2.1. Reactor Set-Up and Operation

**Figure 1:**
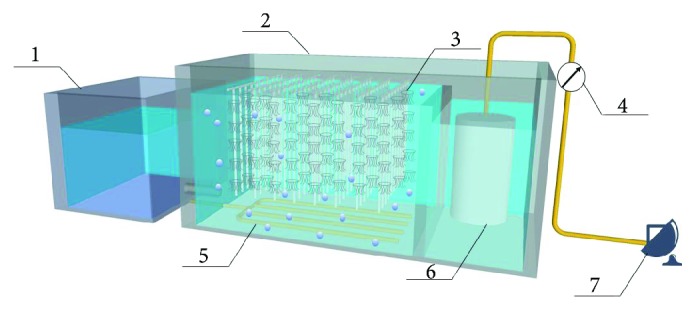
Schematic of the biofilm-membrane bioreactor. 1: regulating tank; 2: biofilm-membrane bioreactor; 3: biofilm carrier; 4: pressure meter; 5: perforated aeration pipes; 6: membrane module; 7: drainage pump.

The BMBR used in the study was made of steel plates with dimensions of 1.08 m × 0.75 m × 0.6 m resulting in a working volume of 400 L. The reactor was divided into a biofilm zone and a membrane zone by a baffle (Figure 1). Semisoft media were assembled in the biofilm zone with a density of 30%. Membrane zone was equipped with hollow fiber membrane module, controlled by a special valve. The influent fully contacted with semisoft media in the upward flow, then overflow into the membrane area. Perforated aeration pipes with a diameter of 20 mm were installed at the bottom of the reactor. The perforated pipes were connected with an air pump, through which the air was aerated into the wastewater. During the experiments, the reactor was operated in continuous influent-intermittent effluent way. Effluent from a mustard WWTP was collected in the regulating tank and then pumped into the reactor. The raw water consisted of 2~3% salinity, 770~1240 mg/L COD, 103~191 mg/L NH_4_^+^-N, 207~409 mg/L TN, 21~48 mg/L phosphate, and 237~525 mg/L SS. The drainage pump worked in an intermittent mode and controlled by a PLC automatic system. The operating cycle of drainage pump was set to be 13 min in total including a 10 min uptake time and a 3 min off time. The membrane flux was measured by a liquid flowmeter. The pressure difference between inside and outside membrane was measured by a negative pressure meter.

The BMBR was firstly inoculated with the sludge from aerobic reactor in the Fuling WWTP and keep the mixed liquor suspended solids (MLSS) above 5 g/L. The reactor was operated continuously under different organic load. The operation of BMBR can be divided into three periods with a corresponding organic load of 0.5 kg COD/(m^3^·d), 1.0 kg COD/(m^3^·d), and 1.5 kg COD/(m^3^·d). During a 110 days operation time period, the aeration intensity was kept at 0.8m^3^/h, and a transmembrane pressure difference (TMP) was remained at 15Kpa. Membranes were cleaned chemically or physically in a way described below. The operating condition and organic load are summarized in Table 1.

### 2.2. Analytical Methods

Samples of influent and effluent were collected from the reactor and analyzed immediately. The following parameters including chemical oxygen demand (COD), suspended solid (SS), ammonium, total nitrogen, and dissolved phosphate were measured according to APHA Standard Methods. DO and pH were measured by a DO detector (HACH, HQ30d, USA) and a pH detector (HACH, sension2, USA), respectively.

### 2.3. Membrane Fouling Analysis and Cleaning

The membrane module was firstly taken out of the reactor and then was scrubbed softly with a sponge under tap water. Physical cleaning was performed to restore the membrane flux by removing the cake layer from the membrane surface. After that, chemical cleaning was carried out to further improve the membrane flux. The membrane module was soaked in NaClO solution (0.5%, m/m) for 24 h and then soaked in tap water for 2 h.

The attachment of membrane was determined by scanning electron microscopy (SEM; Hitachi S-3400N, Hitachinaka, Japan) to get an additional visual insight into the deposition on the surface of membrane.

## 3. Results and Discussion

### 3.1. Reactor Performance

#### 3.1.1. COD Removal Efficiency

**Figure 2:**
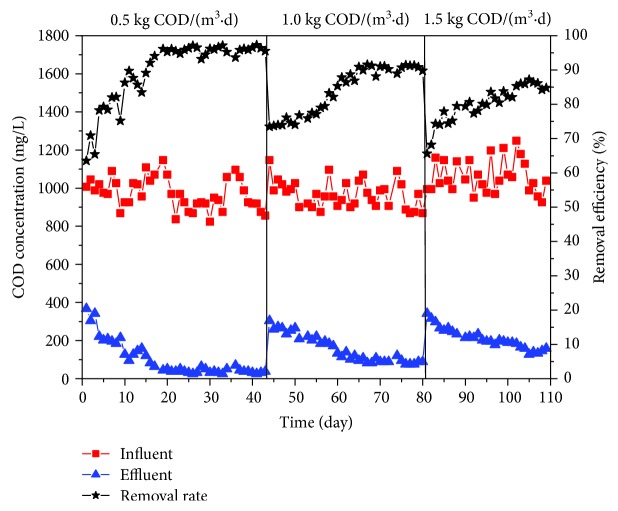
COD concentration variations in influent and effluent.

The COD of influent and effluent over the 110-day operation time period is shown in Figure 2. In stage I, the average COD removal efficiency was greater than 94% with an average COD value of 48.18 mg/L. In stage II, when the influent organic load was 1.0 kg COD/(m^3^·d), the average COD removal rate decreased from 94.81% to 89.35%. In stage III, with the increase of organic load, the average COD removal rate furtherly decrease to 84.90% with average COD of 155.46 mg/L. The existence of a short adaptation period in the beginning of each stage was observed, indicating that the high salinity and organic load had a negative impact on microbes' growth [19]. Due to the application of biofilm and membrane process, the sludge was retained and immobilized in the reactor so that the biomass increased quickly in a short period. After the adaptation period, the COD removal efficiency stayed stable at a high level (84.90%~94.81%). Such a result confirmed the effectiveness and robustness of the biofilm-membrane bioreactor system even in a high organic pollution and salinity level [18]. However, with the increase of organic load from 0.5 kg COD/(m^3^·d) to 1.5 kg COD/(m^3^·d), the trend of COD removal rate started to decline. One possible explanation is that the balance between the microbial degradation ability and pollution loading has been reached when the organic load was below 1.0 kg COD/(m^3^·d). Additionally, the deficiency of dissolved oxygen may hinder the reactivity and growth of microbes because the aeration intensity was kept constant at all stages. Hence, a greater aeration intensity is needed to improve the COD removal efficiency at such a high organic load. Although the COD removal efficiency dropped with the increase of organic load, BMBR still exhibited a great performance and salinity tolerance comparing with the conventional MBRs [20]. Mannina et al. reported that when the feeding salt rate up to 20 g/L, the total COD removal rate decreased from 96% to 75% at an influent COD concentration of 350 mg/L [21].

#### 3.1.2. NH_4_^+^ Removal Efficiency

**Figure 3:**
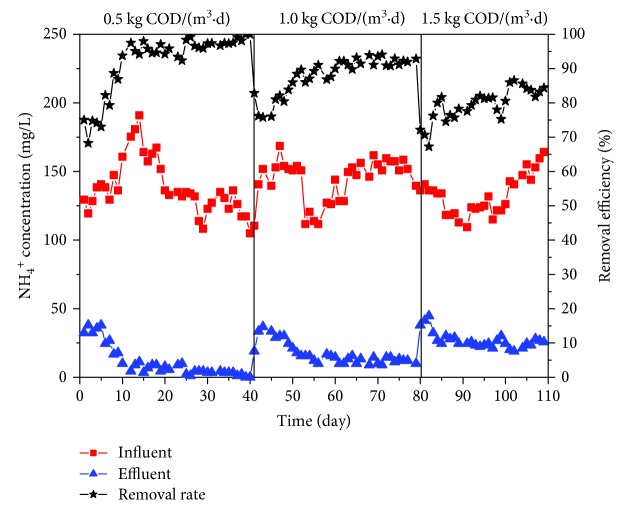
NH_4_^+^-N concentration variations in influent and effluent.

Membrane played an important role in the NH_4_^+^ removal. From Figure 3, the performance of NH_4_^+^ removal was achieved at high level, with a mean removal rate of 96.84% in stage I and 91.26% in stage II. Since nitrifying bacteria are autotrophic bacteria, a longer sludge retention time (SRT) is required for them to reproduce. The function of membrane filtration makes the SRT as long as possible, in which way the nitrifying bacteria accumulated and nitrification enhanced. It should be noted that the NH_4_^+^ removal efficiency has not been influenced greatly when organic load increased from 0.5 to 1.0 kg COD/(m^3^·d). This reflects biofilm in BMBR can improve the impact resistance of the system [22]. However, when the organic load increased from 0.5 to 1.5 kg COD/(m^3^·d), the NH_4_^+^ removal rate sharply dropped by 13.72%. Oxygen availability is one of the most important factors in the nitrification process for nitrifying bacteria. Under the condition that influent COD concentration was up to 1054.29 mg/L, nitrifying bacteria were inferior to other heterotrophic bacteria in the competition for dissolved oxygen, resulting in the reduction of NH_4_^+^ removal efficiency. On the other hand, high salinity may exert inhibition on the nitrification process [23]. Previous studies have confirmed that high salinity negatively affected the transport of nutrient from medium to the cell, consequently modifying and reducing cell metabolism that lead to cell lysis [24]. Zhao et al. discovered that when salt concentration was above 20 g/L, NH_4_^+^ removal efficiency decreased, and the bioreactor collapsed [5].

#### 3.1.3. TN Removal Efficiency

**Figure 4:**
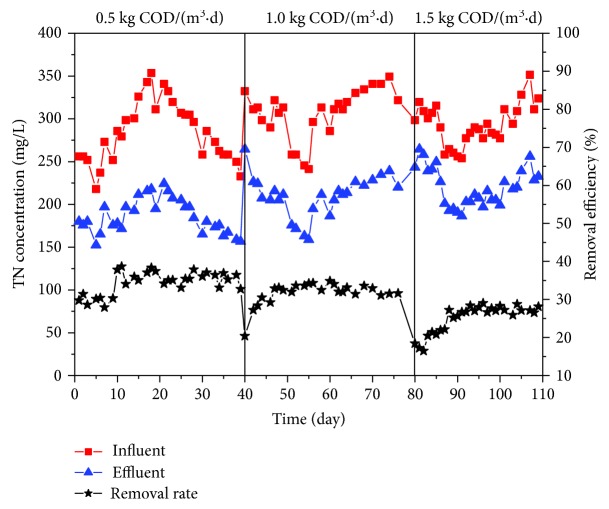
TN concentration variations in influent and effluent.

Fluctuations in TN removal efficiency were observed (Figure 4). The overall TN removal efficiency was relatively low comparing to previous studies. The main reason for the poor TN removal rate was the lack of an anoxic environment for denitrification [25]. Excessive dissolved oxygen made denitrifying bacteria switch from anaerobic to aerobic metabolism so that denitrification was inhibited. There was a general trend of decreasing TN removal as organic load increased from 0.5 to 1.5 kg COD/(m^3^·d). This decrease may attribute to the incomplete nitrification. It has been proved that nitrification is crucial to stimulate TN removal because nitrification can provide nitrate or nitrite needed in denitrification. Although there are multiple novel nitrogen removal paths, e.g., partial nitrification-denitrification, ammonium oxidation [26], nitrification is the first step in nitrogen removal. Therefore, with the decrease of NH_4_^+^ removal efficiency, TN removal rate declined accordingly. Apart from oxygen and nitrification, another important factor that influenced denitrification was salinity. Denitrifying bacteria are more sensitive to toxic substance than nitrifying bacteria [27]. It is detrimental for the growth of denitrifying bacteria in high-salinity environment.

#### 3.1.4. Soluble PO_4_^3−^ Removal Efficiency

**Figure 5:**
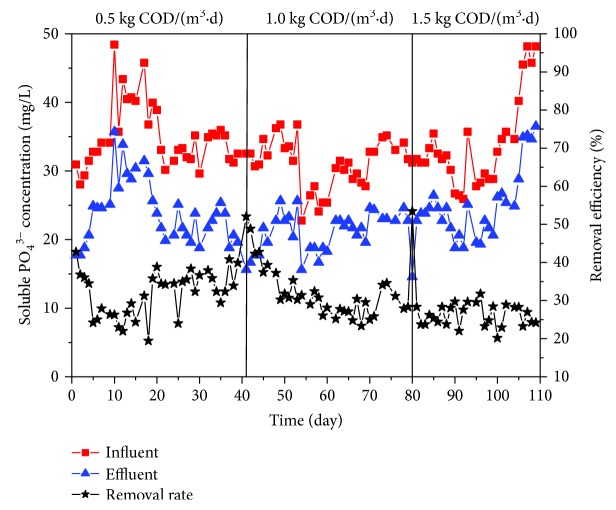
PO_4_^3−^ concentration variations in influent and effluent.

Suspended solids and particle-associated phosphorus could be captured via membrane filtration. In this study, focus was put on the removal efficiency of soluble phosphorus in the BMBR. During the experiment, the general PO_4_^3−^ removal efficiency was poor with significant fluctuations (Figure 5), ranging from 19.23% to 53.89%, which reflected similar results when comparing with other studies [28, 29]. Biological phosphorus removal includes two steps: anaerobic phosphorus release and aerobic phosphorus uptake. However, there was no anaerobic environment available in BMBR. Phosphorus removal mainly depended on biological assimilation. Moreover, phosphorus-rich sludge cannot discharge the reactor in time, leading to the low PO_4_^3−^ removal efficiency. The high PO_4_^3−^ removal that occurred in the beginning of each stage was observed. This was because the chemical cleaning of the membrane module was performed before the working condition changed so that the membrane module can work under the same condition. Phosphorus-rich sludge adhered to the surface of the membrane was cleaned out, and the microbial biomass suddenly decreased. Consequently, more microorganism proliferated, and phosphorus was stored in microbial cells, in which way PO_4_^3−^ removal efficiency increased temporarily.

### 3.2. The Role of Halophilic Bacteria on Membrane Fouling

**Figure 6:**
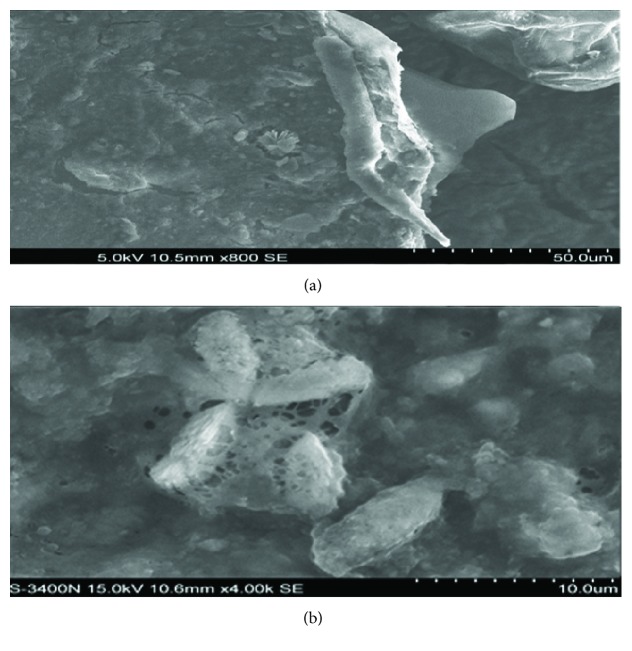
SEM images of membrane fouling. (a) The microorganisms on cake layer; (b) the EPS on gel layer.

**Figure 7:**
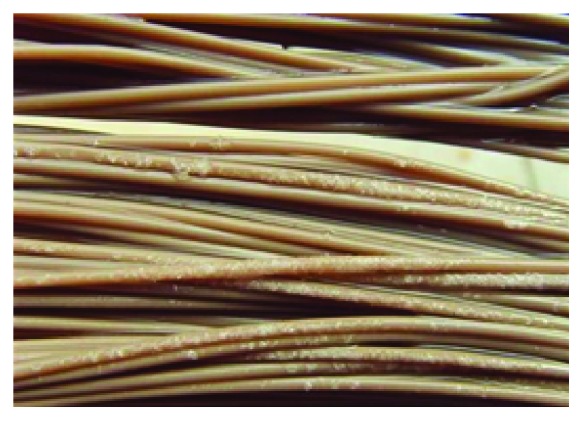
The photograph of NaCl crystallization on the surface of the membrane.

Halophilic bacteria are special microbes that only grow in saline environment. Halophilic can metabolize organic matter and nutrient in the wastewater to gain energy. It is promising to treat high-salinity wastewater using halophilic bacteria [30]. To determine the substance causing membrane fouling, SEM was utilized to analyze the microscopic structure of the membrane pollution (Figure 6). With the increase of influent organic load, halophilic bacteria gradually predominate by succession, characterized by abundance of bacillus and coccus in the reactor. The stabilization of microbial community structure has a beneficial effect on removal efficiency [31, 32]. High salinity also changed the structure and property of sludge [28]. When there is no salt or a low-concentration salt exists, the size of sludge floc is large. However, the sludge floc mainly composed of halophilic bacteria was small and loose [33], which can block the membrane pore and cause irreversible contamination. Some kind of sludge floc attached to the surface of the membrane and formed a gel layer which contained different kinds of extracellular polymeric substance (EPS). Sludge microorganisms secreted EPS to resist adverse saline environment. Hong et al. [34] reported that increasing salt concentration resulted in the rise of EPS concentration. The soluble portion of EPS as well as bound EPS facilitated the formation of the gel layer on the membrane surface [35], which cannot be readily removed by physical cleaning [36]. On the other hand, when the water temperature was below 10°C, the soluble salt recrystallized (Figure 7), contributing to the sharp decrease of membrane flux. Summarily, the membrane fouling was the consequence of the interaction of microbial activities and NaCl crystallization. If the goal is to mitigate the membrane fouling when treating high-salinity wastewater, a low operating temperature should be avoided.

## 4. Conclusion

The biological treatment of mustard tuber wastewater presents to be a great challenge due to the high concentration of organic carbon, nutrient, and salinity that can strongly inhibit microbial activity and damage the settling ability of activated sludge. A novel technology combined with biofilm and membrane bioreactor was developed to treat mustard tuber wastewater. In detail, the microbial biomass can increase quickly in BMBR system because the immobilized biofilm enhances the growth of bacteria. A high removal efficiency of organic carbon and ammonium was achieved indicating that heterotrophic bacteria and nitrifying bacteria maintained high reactivity in the saline environment. However, the removal of total nitrogen and soluble phosphorus was relatively low due to the lack of anaerobic environment. With the increase of influent organic load, the performance of the BMBR degenerated when the organic load exceeded the microbial degradation ability. Halophilic bacteria played a key role in pollutant removal as well as in the biofouling process. Under a low-temperature operation, membrane fouling was the consequent of the interaction of microbial activities and NaCl crystallization. Finally, BMBR system showed a high potentiality in treating high-concentration or high-salinity wastewater.

## Figures and Tables

**Table 1:** Summary of the tested schemes.

Operating condition	Organic load (COD/(m^3^·d))	Average influent concentration (mg/L)				
		COD	NH_4_^+^	TN	PO_4_^3−^	SS
Stage I	0.5	962.44	137.51	284.23	34.92	402.26
Stage II	1.0	959.97	142.92	317.98	30.64	355.90
Stage III	1.5	1054.29	133.64	293.64	33.58	428.46

## Data Availability

The data used to support the findings of this study are available from the corresponding author upon request.
